# Interplay between Neuroendocrine Biomarkers and Gut Microbiota in Dogs Supplemented with Grape Proanthocyanidins: Results of Dietary Intervention Study

**DOI:** 10.3390/ani10030531

**Published:** 2020-03-22

**Authors:** Elisa Scarsella, Michela Cintio, Lucilla Iacumin, Federica Ginaldi, Bruno Stefanon

**Affiliations:** Department of Agriculture, Food, Environmental and Animal Science, University of Udine, 33100 Udine, Italy; scarsella.elisa@spes.uniud.it (E.S.); michela.cintio@uniud.it (M.C.); lucilla.iacumin@uniud.it (L.I.); ginaldi.federica@spes.uniud.it (F.G.)

**Keywords:** proanthocyanidins, fecal microbiota, end products of fermentation, serotonin, cortisol, *Canis lupus familiaris*

## Abstract

**Simple Summary:**

The connection between animal health and gut microbiota has been studied during the past years through different diet modulation experiments; however, there is still a paucity of information about the prebiotic functions in the gastrointestinal tract of companion animals. Considering this, a population of dogs living in the same environment has been subjected to a nutritional study, with different doses of proanthocyanidins extracted from grapevine supplied to the diet. Characterization of the gut microbiota and data from endocrine analysis in saliva have been collected. Dogs responded differently to the dietary intervention, and results underlined the existence of a difference between subjects in terms of fecal microorganisms and neuroendocrine markers, leading us to think the balance of gut microbiota is going to play a strong role in diet formulation based on host health modulation.

**Abstract:**

Several studies on the interaction between gut microbiota and diets, including prebiotics, have been reported in dogs, but no data are available about the effects of dietary administration of grape proanthocyanidins. In the study, 24 healthy adult dogs of different breeds were recruited and divided in 3 groups of 8 subjects each. A group was fed with a control diet (D0), whilst the others were supplemented with 1 (D1) or 3 (D3) mg/kg live weight of grape proanthocyanidins. Samples of feces were collected at the beginning and after 14 and 28 days for microbiota, short chain fatty acid, and lactic acid analysis. Serotonin and cortisol were measured in saliva, collected at the beginning of the study and after 28 days. A significantly higher abundance (*p* < 0.01) of *Enterococcus* and *Adlercreutzia* were observed in D0, whilst *Escherichia* and *Eubacterium* were higher in D1. *Fusobacterium* and *Phascolarctobacterium* were higher (*p* < 0.01) in D3. Salivary serotonin increased (*p* < 0.01) at T28 for D1 and D3 groups but cortisol did not vary. Proanthocyanidins administration influenced the fecal microbiota and neuroendocrine response of dogs, but a high variability of taxa was observed, suggesting a uniqueness and stability of fecal microbiota related to the individual.

## 1. Introduction

The interaction of intestinal microbiota with the host has attracted the scientific community, and a large body of research has been published to highlight the coevolution of anatomical, physiological, immunological, and developmental functions of host and microbiota [1]. Other studies have underpinned the interplay between gut microbes and their products of fermentation with the host, not only from a nutritional point of view [2] but also for the modulation of immunological, endocrinal, and neurological functions [3]. 

The bidirectional link between the host brain and the gut microbiota relies mainly on the neural communication of the central nervous system (CNS) with the periphery and on the humeral communications [4]. Neural communications involve the vagus nerve and the dorsal root ganglia of the small and large intestine, through projections from the enteric nervous system to sympathetic ganglia and parasympathetic innervation of the gut. Humeral communications also depend on products of microbial activity, cytokines, and hormones. 

Among the compounds that are involved in the connection of the emotional and cognitive centers of the brain with the gut and its resident microbiota, serotonin plays a paramount role. Other than being a neurotransmitter within the CNS, serotonin is secreted by the enterochromaffin cells of the intestinal epithelium and stimulates gut motility [4], regulating the transit time of the food and then the extent of bacterial fermentation and the amount of end products of fermentation.

Gut microbiota interacts with tryptophan metabolism and influences its amount available for serotonin synthesis, thus interacting with the serotonergic system [5]. The modification of tryptophan metabolism, reported for instance for irritable bowel syndrome, implies a serotonin deficiency, which leads to a depressive mood, or to the production of neurotoxic/neuroprotective metabolites that have the CNS as target [6]. Furthermore, microbial populations synthesize other signal molecules, such as GABA and short-chain fatty acids (SCFAs), which have effects on the gut epithelium, on the local mucosal immune system, and on the vagus nerve [7]. Perturbation of gut microbiota also induce the modulation of other neurotransmitters and signal molecules (e.g., dopamine, cytokines, interleukins, and Tumor Necrosis Factor (TNF) from the gastrointestinal tract, which can in turn activate a neuroendocrine response, as the hypothalamus adrenal pituitary axis (HPA), or can modulate the metabolism of tryptophan, inferring with the serotonergic system [7]. 

The use of food additives as prebiotic, probiotic, and synbiotic offers a therapeutic approach to restore the gastrointestinal and microbial balance [8], but less information is available for other bioactive compounds, as polyphenols. In plants, polyphenols are in a glycosylate form and after ingestion by the organism they are recognized as xenobiotics; therefore, their catabolized bioavailability is reduced in comparison with the common nutrients [9]. It has been estimated that only 5%–10% of total polyphenol intake is absorbed in the small intestine, while the remaining 90%–95% may be accumulated in the colon lumen where it is consequently processed by the enzymatic activities of the gut microbial population [10]. Therefore, it is likely that microorganisms populating the gut can be involved in the absorption of polyphenolic structure, thanks to the conversion in low-molecular-weight compounds. Consequently, evidence suggests that the health benefits of polyphenols are related not only to the original molecules found in plants but also to their intermediates and end-products, given the greater bioavailability compared to the parent molecules [11]. However, the metabolism of polyphenols has been reported only for few microbial species of the gut [12], which depends on the daily dose and on the individual variability of the gut microbiota community. Hence, inter individual differences in the composition of the gut microbiota may lead to variations of bioavailability and bioactivity of polyphenols metabolites [13]. Nevertheless, recent studies have shown that both polyphenols and polyphenols-derived molecules are able to shape hindgut microbiota through selective prebiotic effects and antimicrobial activities against pathogenic microorganisms [10].

The aim of the study is to evaluate the activity of titrated proanthocyanidins extracted from grape on the gut microbiota of dogs and the relationship with endocrine responses measured in saliva. The study was performed with resident dogs housed in a shelter and fed a standard diet supplemented with two doses of grape proanthocyanidins.

## 2. Materials and Methods

### 2.1. Animals and Housing

Twenty-four healthy adult dogs of different breeds were recruited for the study (Table S1). The group consisted of 18 castrated and 3 intact males and 3 spayed females. Dogs were housed in the same shelter and allocated in pairs in different boxes of 6 × 3 m enclosures, with a 2 × 3 m roof covering the paved portion of the pen and equipped with beds. Dogs were fed in the box at around 16:00 p.m., and water was always available. The study started in June in Northeast Italy (46.029051 N; 13.231521 E), with an average temperature during the period of 20–30 °C and 60%–70% relative humidity. At the beginning of the study, dogs were weighed and the average live weight was 28 ± 9 kg.

All protocols, procedures, and the care of the animals complied with the Italian legislation on animal care (DL n.116, 27/1/1992) and the study was approved by the ethical committee of the University of Udine.

### 2.2. Experimental Design

From at least 90 days from the beginning of the study and during the period, dogs were fed commercial extruded diet, formulated with beef meat, cereals and potato, chicken fat, beet pulp, flax seeds, salmon oil, yeast, minerals, and vitamins. The chemical composition was 90% dry matter, 26.2% crude protein, 15.6% crude lipid, 2.3% crude fiber, and 9.1% ash. 

The recruited dogs did not receive antibiotic treatments or probiotic supplementations, at least two months before the experiment started. Moreover, before the beginning of the study, dogs were divided in 3 groups of 8 individuals each, matched for live weight and including 1 female per group. During the study, dogs were housed in the usual boxes. The first group received placebo tablets without polyphenols (D0), whilst the second group and the third group were supplemented with tablets containing 1 mg/kg of live weight (D1) or 3 mg/kg of live weight (D3) of dried extract of grape polyphenols (ARDA Natura, Fiorenzuola D’Arda, PC, Italy), standardized to >95% of proanthocyanidins. The D0, D1, and D3 tablets were produced by Tecnozoo s.r.l. (Torreselle di Piombino Dese, Padova Italy), with barley malt extract, dextrose, sucrose, magnesium stearate, and E554 (sodium silicate and synthetic aluminum) as additives.

### 2.3. Collection of Samples

For both the studies, stool and saliva samples were collected before the meal at the beginning of treatment (T0), after 14 days (T14), and at the end of the trial, after 28 days (T28). The feces were collected after evacuation from the ground using sterile gloves and were placed in 50 mL sterile tubes and immediately frozen at −20 °C until analysis. Salivary samples were collected with SalivaBio swabs (Salimetrics, LLC 101 Innovation Boulevard, State College, PA, US) following the procedure previously described [14]. After sampling, the swabs were introduced into tubes specifically designed to avoid cortisol sequestration (Salivette; no. 51.1534, Sarstedt, Nümbrecht, Germany), temporally stored in an iced box before the final storage at −20 °C, until analysis. The hair samples were collected from the neck of the dogs at the beginning of the study and the regrowth was sampled after 28 days. Hair samples were introduced in a paper envelop to avoid condensation and stored at room temperature until analysis.

### 2.4. Short Chain Fatty Acids and Lactic Acid Analysis in Feces

The analysis of lactic acid and short chain fatty acids (SCFAs) (acetic; propionic; butyric) were performed by HPLC according to the following procedure: 1 g of fecal material was diluted with 50 mL of 0.1 N H_2_SO_4_ aqueous solution and homogenized for 15 min by a mechanical stirrer (Instruments Srl, Milano, Italia). The mix was centrifuged (20000× *g* for 20 min at 4 °C) to separate the liquid phase from the solid residuals, and the liquid phase was subsequently microfiltered with 0.45 µm syringe filter of polypore (Alltech, Casalecchio di Reno BO, Italy). A total of 20 µL of the resulting sample was directly injected in the HPLC instrument using an Aminex HPX-87H ion exclusion column (300 mm × 7.8 mm, 9 µm) and a precolumn (Bio-Rad, Hercules, CA, USA) kept at 40 °C. The isocratic elution flux was 0.6 mL/min and using 0.008 N H_2_SO_4_ solution as a mobile phase the detection length was 220 nm. The concentration of SCFAs (acetic, propionic, butyric, isobutyric, valeric and isovaleric) and lactic acid of fecal samples was measured by HPLC according to the protocol described by Sandri et al. (2017) [15]. SCFAs and lactic acid concentrations were calculated with reference to a standard solution of 4.50 mg/mL of lactic acid, 5.40 mg/mL of acetic acid, 5.76 mg/mL of propionic acid, 7.02 mg/mL of butyric acid and isobutyric acid, 8.28 mg/mL of valeric acid, and isovaleric acid in 0.1 N H_2_SO_4_ (Sigma–Aldrich^®^ Co., Milan, Italy). Quantifications were calculated using an external calibration curve based on these standards. The sum of SCFAs and lactic acid was calculated and the single acid was expressed as molar percentage of the total acids (TA). 

### 2.5. Fecal DNA Extraction, Sequencing, and Taxonomic Annotation

Microbial DNA from fecal samples was extracted from 150 mg of starting material using a Fecal DNA MiniPrep kit (Zymo Research; Irvine, CA, US), following the manufacturer’s instruction, including a bead beating step. DNA concentration was measured with a QubitTM 3 Fluorometere (Thermo Scientific; Waltham, MA, USA), then DNA was fragmented and the 16S rRNA of V3 and V4 regions amplified for library preparation, adding also the Indexes for sequencing, using a Nextera DNA Library Prep kit (Illumina; San Diego, CA, USA), following manufacturer’s instruction and primers [16]. The resulting amplicons were sequenced with a MiSeq (Illumina; San Diego, CA, USA) in 2 × 300 paired-end mode, following the standard procedures. 

The Quantitative Insights into Microbial Ecology (QIIME 2) [17] was used to process the raw sequences, which were uploaded to NCBI Sequence Read Archive (Bioproject ID PRJNA564012). After demultiplexing, sequenced reads that passed the quality check (Phred score ≥30) were annotated for 16S rRNA against the most recent Greengenes database (version gg.13_8.otus.tar.gz), with 99% identifying with reference sequences. Chimeras were also detected and then filtered from the reads, and the remaining sequences were clustered into exact sequence variants by using an open reference approach in QIIME 2. This procedure is the preferred strategy for exact sequence variants picking in QIIME2, which includes taxonomy assignment, sequence alignment, and tree-building steps.

### 2.6. Quantitative Real-time PCR (qPCR)

Quantification of total bacteria and of taxa used to describe dysbiosis index in feces, namely, *Faecalibacterium* spp., *Fusobacteria*, *Blautia* spp., *Turicibacter* spp., *Escherichia coli*, *Clostridium hiranonis*, and *Streptococcus* spp., were evaluated by qPCR using the oligonucleotides tested by AlShawaqfeh et al. (2017) [18].

The qPCR data for *Faecalibacterium* spp., *Fusobacteria*, *Blautia* spp., *Turicibacter* spp., *Escherichia coli*, *Clostridium hiranonis*, and *Streptococcus* spp were normalized to the qPCR data for total bacteria and therefore expressed as percentage of the total bacteria. All samples were run in triplicate.

SYBR-based qPCR assays were performed following the run protocol reported by AlShawaqfeh et al. (2017) [18] with some modifications. Briefly, SYBR-based reaction mixtures (total 10 μL) contained 5 μL of SsoFast^TM^ EvaGreen^®^ supermix (BioradLaboratories, US), 1.6 μL of water, 0.4 μL of each primer (final concentration: 400 nM), and 2 μL of DNA previously standardized at 25 ng/μL. PCR conditions were 95 °C for 2 min, and 40 cycles at 95 °C for 5 and 10 s at the optimized annealing temperature. A melt curve analysis was performed for SYBR-based qPCR assays under the following conditions: 1 min at 95 °C, 1 min at 55 °C, and 80 cycles of 0.5 °C increments (10 sec each). A RotorGene Q thermal cycler (Qiagen, Germany) was used for all qPCR assays. Data are expressed as average values and standard deviations.

### 2.7. Endocrine Analysis of Saliva and Hair Samples

For cortisol analysis in the hair, the method described by Accorsi et al. (2008) [19] was used, with minor modifications [20]. Briefly, 150 mg of hair were weighted from each sample and placed into 15 mL glass vial. Samples were washed three times with 2.5 mL of isopropanol (2-propanol 99.5%, Sigma Aldrich, Milan, Italy) and 3 min of vortex. Isopropanol was discarded after each wash, and after the final wash, hair samples were placed on a plastic disk and let dry for 48 hours at room temperature. Dried hair samples were trimmed with a blade, and 50 mg of trimmed hair were weighed and placed into a 15 mL glass centrifuge tube with 5 ml of methanol. Samples were incubated in water bath at 45 °C for 18 h under moderate shaking. At the end of incubation, tubes were centrifuged at 5000 g for 10 min, and 2 mL of supernatant was transferred to a 1.5 mL Eppendorf tube and centrifuged in a spin-vacuum (Centrifugal System, RC 10.10, Jouan, Cologno Monzese, Italy) at 40 °C until complete evaporation of methanol. Dried samples were then reconstituted with 0.6 mL of PBS, with 0.1 % bovine serum albumin (Sigma Aldrich, Milan, Italy). Salivary swabs were thawed and centrifuged at room temperature at 1500 g for 15 min to obtain clear saliva for the analysis. 

Cortisol concentrations in saliva (HCS) and hair (HCH) samples were measured according to the RIA procedure, as described by Sgorlon et al. (2015) [21]. Samples were assayed in duplicate, the sensitivity of the assay was 3.125 pg/well, and the intra-assay and inter-assay coefficients of variation in high and low cortisol reference samples were 5.9% and 9.1% and 13.5 % and 15.1 %, respectively.

Serotonin was determined in salivary samples (SES) with an ultrasensitive enzyme immunoassay commercial kit (Serotonin Research ELISA DEE5900; Demeditec Diagnostic Gmbh Germany), designed to measure serotonin in various biological samples. Samples were assayed in duplicate, and the sensitivity of the test was 0.005 ng/mL and specificity (cross reactivity) was 100% for serotonin, 0.19% for tryptamine, and lower than 0.03% for other related compounds. The intra-assay and inter-assay coefficients of variation in high and low serotonin reference samples were 7.0% and 9.9% and 16.9 % and 18.1%, respectively. 

### 2.8. Computation and Statistical Analysis

Data were imputed on a spreadsheet for analysis. The 16S rRNA annotated sequences were normalized to ‰ abundance profiles for each sample and each taxonomic level. Taxa with relative abundance lower than 10‰ [22,23,24] in more than half of the samples were excluded from the statistical analysis. The average percentage of reads excluded was 1.9%. Shannon α-biodiversity index was calculated at the genus level including all taxa according to the equation H’ = −sum(Pi × ln Pi), where Pi = frequency of every genus within the sample. Evenness index was calculated as J’ = H’ / ln S, where S = total number of genera within each sample. Beta diversity was evaluated with the phylogeny based UniFrac distance metric [25] and visualized using principal coordinate analysis (PCoA) plots. 

Analysis of similarity (ANOSIM) was then performed to test whether the microbial communities differed significantly between D0, D1, and D3 diets at T0, T14, and T28 times of sampling, using the ‘Vegan’ package in R (Version 3.2.1). A linear discriminant analysis (LDA) effect size (LEfSe) was applied to detect taxa that differed between D0, D1, and D3 groups at T0, T14, and T28 [26]. 

Before statistical analysis, normality of distribution of the independent variables was checked with the nonparametric Kolmogorov–Smirnoff test. Linear Mixed Model was used to analyze the SCFA and lactic acid concentrations, their molar proportions, and H’ and J’ indexes. The model included the fixed effect of time of sampling (3 levels, T0, T14, and T28), treatments (3 levels D0, D1, and D3), and the interaction of time of sampling with treatment, with the subject (dog) as random factor repeated over the time of sampling. For cortisol (HCS) and serotonin (SES) in saliva and cortisol in hair (HCH), the same model was used, using two levels for the fixed effect of time of sampling (T0 and T28). Data obtained by qPCR analysis were subjected to two-way ANOVA to test the data obtained during the time for each microbial group. If appropriate, means were compared by Tukey’s multiple range test for *p* < 0.05. Statistical analysis were performed with XLSTAT [27]. 

## 3. Results

The mean concentration of lactate and SCFAs in the fecal samples of the dogs are reported in Table 1. Molar content of acetate, butyrate, isobutyrate, and isovalerate did not significantly differ between diets and time of sampling, whilst for propionate a significant increase (*p* < 0.05) was observed at T28 for the D3 group, and a significant (*p* < 0.05) interaction of diet with time of sampling was calculated for lactate. The latter was related to an exceptional high concentration in the sample of a single dog at T0 of the D3 diet, even though no gastrointestinal disease or other symptoms were observed. Looking at the molar proportion of SCFAs and lactate, a significant effect was observed also for isobutyrate, which was higher (*p* < 0.05) at T28 for D0 diet. 

**Figure 1:**
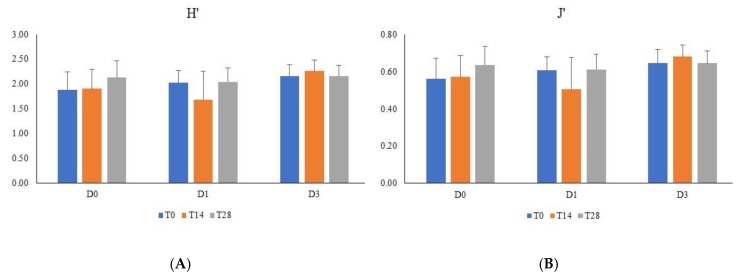
(**A**) Shannon index of biodiversity (H’) and (**B**) Evenness (J’) of the microbial communities for dietary treatments with increasing dose of grape proanthocyanidins. H’ and J’ were calculated on the relative abundances of genera in the feces of dogs fed a basal diet supplemented with increasing amount of grape proanthocyanidins (D0, D1, and D3). D0: Dogs without supplementation of proanthocyanidins; D1: Dogs supplemented with 1 mg/kg live weight of proanthocyanidins; D3: Dogs supplemented with 3 mg/kg live weight of proanthocyanidins. T0: beginning of the study; T14: after 14 days of the study; and T28: after 28 days of the study.

**Figure 2:**
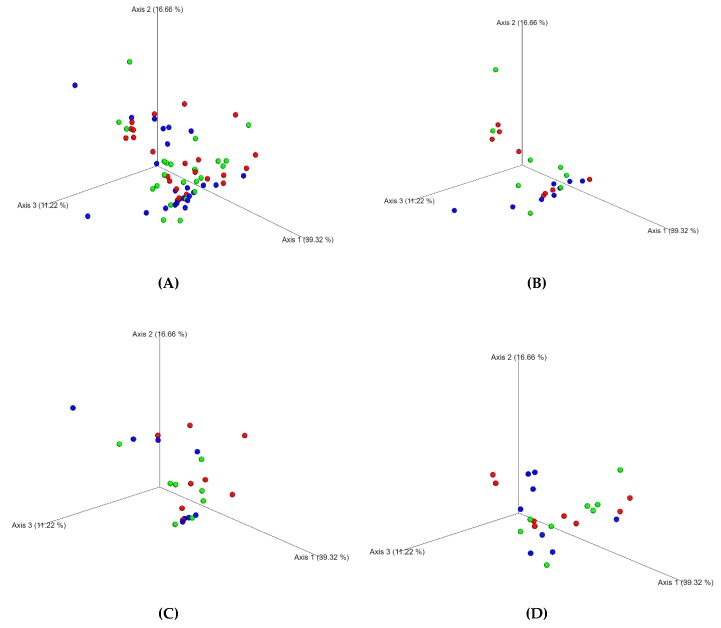
Principal coordinate analysis (PCoA) plot representing the beta diversity of the microbial community between dietary treatments with increasing dose of grape proanthocyanidins (D0, D1, and D3). PCoA was calculated on the weighted UniFrac distance matrices. (**A**) Beta diversity for the three dietary treatments and the three times of sampling; (**B**) beta diversity for the three dietary treatments and at the beginning of the study (T0); (**C**) beta diversity for the three dietary treatments after 14 days (T14); and (**D**) beta diversity for the three dietary treatments after 14 days (T14).

The effect of proanthocyanidins administration to the dogs on fecal microbiota was initially evaluated in terms of biodiversity. The Shannon index of alpha biodiversity (H’) and the derived Evenness index (J’), calculated on the relative abundances of microbial genera in the feces, did not significantly differ between the times of sampling (T0, T14, and T28) and the treatments (D0, D1, and D3) (Figure 1). The principal coordinate analysis, calculated on the weighted UniFrac distance matrices, was employed to assess the beta diversity of the microbial community (Figure 2) between dietary treatments (D0, D1, and D3) and times of sampling (T0, T14, and T28). The analysis of data with ANOSIM, analyzed for each of the three times of sampling or all together, did not significantly differ between dietary treatments, and it was not possible to identify cluster of dogs on the basis of dietary treatments or times of sampling.

**Figure 3:**
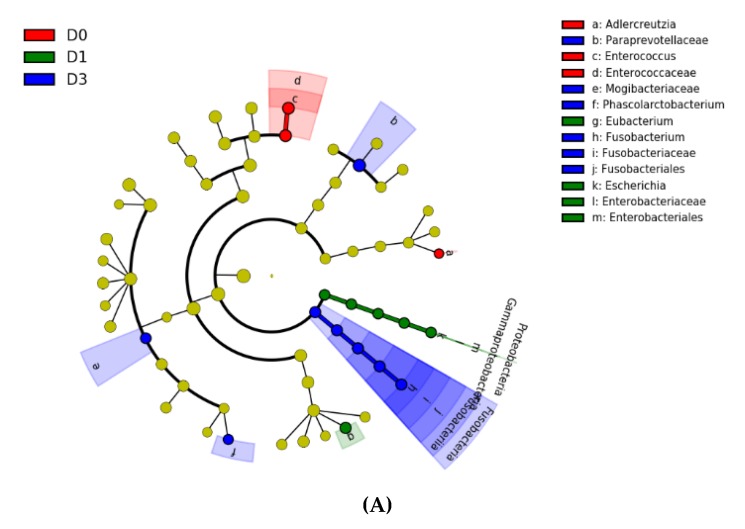

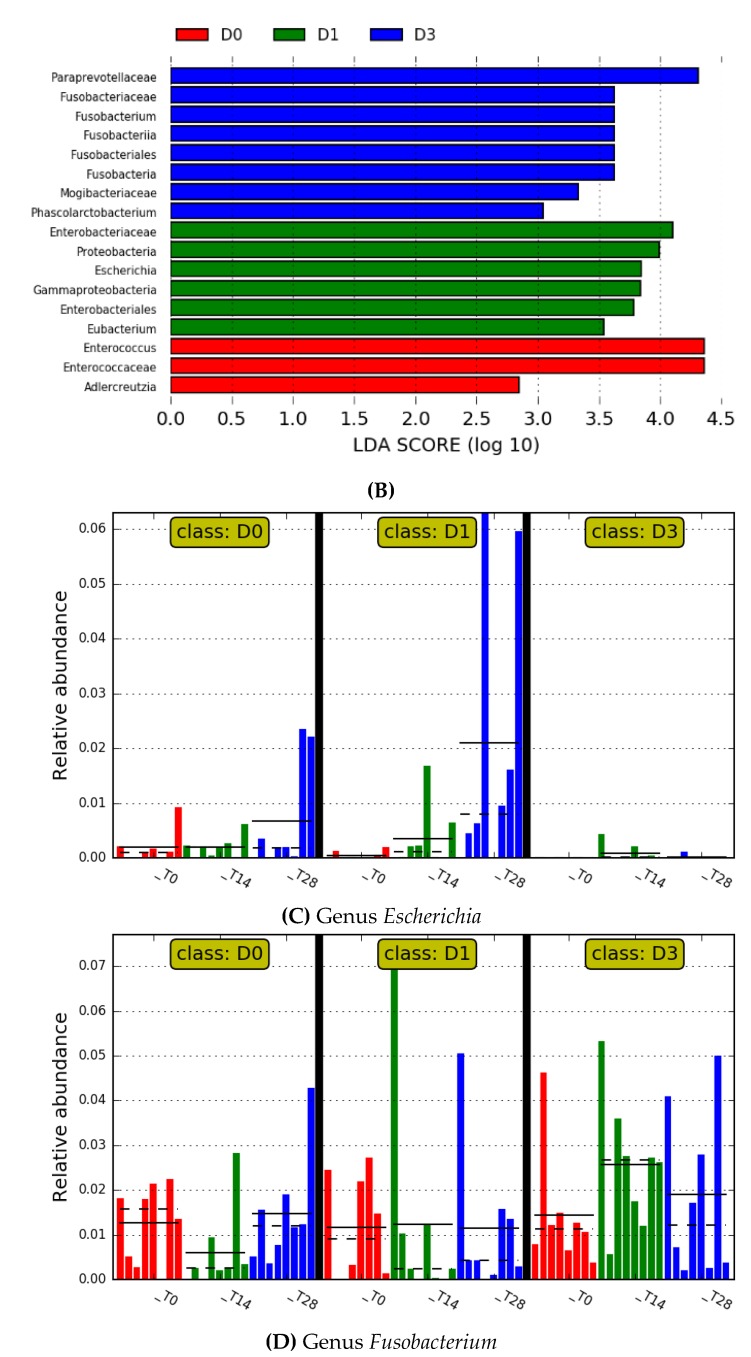
Bacterial taxa differentially abundant in the feces of the dogs without administration of proanthocyanidins (D0), receiving a supplementation of 1 mg/kg live weight (D1) or 3 mg/kg live weight (D3) of proanthocyanidins. The cladogram in (**A**) highlights impactful communities within each treatment and (**B**) shows the score of the linear discriminant analysis (LDA, significant threshold > 2). (**C**) and (**D**) show the individual data for two of significant genera, where dotted line denotes the median and solid line the mean of each subgroup.

The effect of proanthocyanidins on fecal microbiota is depicted in Figure 3, which reports the results from LEfSe analysis. The cladogram (Figure 3) highlights taxa that were significantly affected by dietary treatments (Figure 3A) and the increase of significant relative abundance is also reported (Figure 3B). The relative abundance of the family Enterococcaceae and its genus *Enterococcus*, together with genus *Adlercreutzia*, were significantly higher in subjects fed with D0 diet. The relative abundance of family Enterobacteriaceae representing genus *Escherichia* and genus *Eubacterium* were the most abundant taxa in dogs with D1 treatment. Finally, for the D3 diet, the relative abundances of families Paraprevotellaceae, Mogibacteriaceae and Fusobacteriaceae and of genera *Fusobacterium* and *Phascolarctobacterium* were significantly higher. The relative abundance of genera, which significantly differed between the three treatments (Figure 3C,D) showed a high individual variability. Genus *Fusobacterium* had a significant increase at T14 and T28 for the subjects fed with D3 diet, and genus *Escherichia* showed a higher relative abundance at T28 of D1 diet.

Serotonin (SES) and cortisol (HCS) were measured in saliva and the latter also in hair (HCH) at the beginning and at the end of the study. The mean value of cortisol in hair and saliva (HCS and HCH, respectively) and of SES in saliva at T0 and T28 are reported in Table 2, together with HCS:SES and HCS:HCH ratios. Time of sampling caused a significant increase of HCS and SES, whilst HCH did not vary. However, a consistent increase (*p* < 0.01) of SES was observed at T28 for the D1 and D3 diets. Diet and time of sampling significantly (*p* < 0.05) affected the ratio of HCS:SES, and time of sampling significantly influenced the ratio of HCS:HCH (*p* < 0.01).

**Figure 4:**
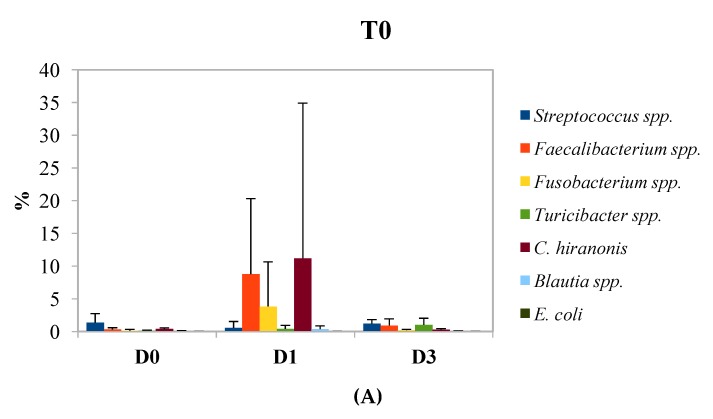

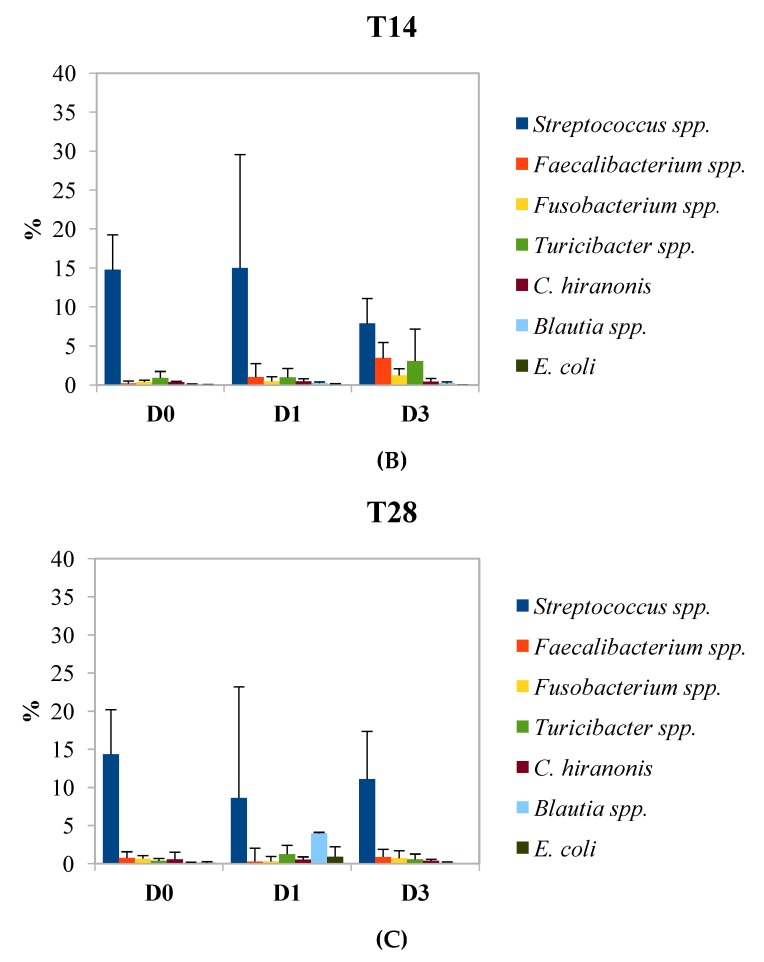
Results obtained from the analysis of the qPCR data. Each panel contains the quantification of the microbial communities researched in the feces of the dogs fed with increasing amounts of proanthocyanidins (D0, D1, and D3). D0: 0 mg/kg live weigh of grape proanthocyanidins; D1: 1 mg/kg live weight of grape proanthocyanidins; and D3: 3 mg/kg live weight of grape proanthocyanidins. (**A**) T0: beginning of the study; (**B**) T14 after 14 days of the study; and (**C**) T28: after 28 days of the study.

The results of the qPCR analysis are reported in Figure 4. The panels A, B, and C represent the percentage of *Faecalibacterium* spp., *Fusobacteria*, *Blautia* spp., *Turicibacter* spp., *Escherichia coli*, *Clostridium hiranonis*, and *Streptococcus* spp. during time in comparison of total bacteria. At the beginning of the experiment (Figure 4A), the amount of the monitored microorganisms was similar in group D0 and D3, while in group D1, a higher amount of *Faecalibacterium* spp., *Fusobacterium*, and *Clostridium hiranonis* was found, corresponding to the 8.8%, 3.8%, and 11.2%, respectively. After 14 days of the treatment (Figure 4B), several changes have been observed. For the D0 group, the concentration of *Streptococcus* spp. increased considerably, as in the other groups, reaching a concentration of about 15% of the total quantified species (D0 and D1) and 7.9% in D3 samples. Compared to T0, the amount of *Faecalibacterium* spp., *Fusobacterium* spp., and *Clostridium hiranonis* decreased significantly (*p* < 0.05). In group D3, there was an increase in the amount of the analyzed microorganisms. The last sampling point is characterized by a slight decrease of *Streptococcus* spp. in all the treatments, but only in group D3 this reduction was significant. Moreover, the other microorganisms decreased among groups except for *Blautia* spp. in group D1, where a significant increase was observed. *Escherichia coli* was detected in a very low amount in all the tested groups. Despite this, it is possible to observe that, at T28, for D0 and D1 treatments, the percentage of *Escherichia coli* was higher than in T0, while in the D3 group, it was lower. In fact, at the beginning of the experiment (T0), the abundance of *Escherichia coli* was equal to 0.023%, 0.007%, and 0.003% for the groups D0, D1, and D3, respectively, whereas, after 28 days, it was 0.117%; 0.933%; and 0.002% for the groups D0, D1, and D3, respectively.

## 4. Discussion

Recently, many researchers have focused their attention on studying the effects that prebiotics, probiotics, or synbiotics can have on the gut microbiota [24,28,29]. Among the compounds that could have the ability to affect intestinal microbial communities, polyphenols have gained popularity. However, there are still relatively few researchers who have tested the potential effects of different polyphenols sources on mice [30] and on humans [31]. To the best of our knowledge, limited studies have analyzed the influence that bioactive compounds can exert on the gut-brain-microbiota axis in dogs. The purpose of this study was to investigate the relationship between polyphenols administration with gut microbial community, end products of fermentations, and endocrine biomarkers, validating the brain-gut-microbiota axis also in dogs.

In the present study, proanthocyanidins were supplemented with the tablets, which corresponded to a concentration of 71 and 203 mg/kg of kibble. In a study of Fragua et al. (2017) [32], the effect of dietary supplementations of 240 and 480 mg/kg kibble of a grape and blueberry extract on working memory in aged dogs was investigated. These authors reported that, after 75 days of supplementation, a significant improvement of cognitive response of dogs was observed for both the amounts in comparison to the control group. Considering that the extract contained 27% of polyphenols, the concentration of these active compounds was 65 and 130 mg/kg kibble.

The average concentration of SCFAs in the fecal samples (Table 1) was 272.4 µmol/g and was higher than the values reported for dogs fed diets with low fiber content [33], which were in a range of 195.5–216.9 µmol/g. In adult dogs, SCFAs supply only 2% to 7% of the maintenance energy requirements [34]. Therefore, despite the fact that they do not represent the major source of energy, as for ruminants, it has been demonstrated that SCFAs can improve gut health by reducing the production of cytokine or other inflammatory molecules within the intestinal mucosa [7]. 

Considering the data of SCFAs and lactic acid (Table 1), it was interesting to note that for the D3 samples, the mean molar proportions of propionate and isobutyrate were higher and lower, respectively, for the D3 diet at T28. The concentration of acetate and propionate are positively related to the amount of fiber in the diet [35] and that of branched chain fatty acids (isobutyrate and isovalerate) are more related to amino acids metabolism in the gut [20]. In the present study, the diet was the same for all the dogs and the only variation was the administration of proanthocyanidins and, accordingly, the observed change of SCFAs could be related to a shift of microbial community. 

At first sight, the microbiota were not modified by the administration of proanthocyanidins, since the biodiversity index H’ and Evenness J’ (Figure 1) and beta diversity (Figure 2) did not significantly change. As reported by Suchodolski et al. (2012) [36], a significant decrease of biodiversity is associated to inflammatory bowel disease in dogs. Moreover, it has been reported that there is a relationship between the low biodiversity of intestinal microbiota and high microbial fitness, with subsequent unhealthy eating behavior and obesity of the subjects [37]. In healthy dogs, as those recruited for this study, changes of biodiversity can be probably detected when the variation of nutrients supplied with the diet is relevant [22]. 

The comparison of relative abundances between groups showed for the D3 group a significant shift of some taxa (Figure 3). Since the diet was the same for all the dogs, the observed changes of relative abundance could be attributed to the administration of proanthocyanidins. Polyphenols are considered safe for dog nutrition [38] and were studied for their antioxidant properties, to prevent or support therapy for arthritis [39,40,41] or to increase cognitive ability [32]. However, a literature search did not produce any results reporting the effect of proanthocyanidins on fecal microbiota of dogs. Jose et al. (2017) [42] showed that the administration of polyphenols from pomegranate peel to dogs caused a shift of fecal pH and ammonia and lactate concentrations, suggesting a positive impact on gut fermentation, but no data on microbial population was reported. In another study, the dietary supplementation of eugenol to dogs led to a reduction of pH and ammonia in the feces, a decrease of *Parabacteroides*, and an increase of *Megamonas*. In humans, the consumption of red wine polyphenols [43] caused an increase of Fusobacteria in the gut, and in rats, the administration of grape seed proanthocyanidins influenced gut microbiota and caused an increase of *Phascolarctobacterium*, other than other bacteria genera. In swine [44], the correlations between microbial taxa and phenolic acids end products were investigated using network analysis. The authors reported that epicatechin catechin, the monomeric units of proanthocyanidins, were positively correlated with the Mogibacteriaceae family. These data would corroborate our findings and, thus, some bioactivity of proanthocyanidins on fecal microbiota of dogs. However, these results must be considered with caution since the taxa significantly different for the D1 did not correspond to those found for the D3 group or, in any case, no dose response effect was observed. Moreover, in Figure 3, the individual relative abundances of 2 significant taxa at T0, T14, and T28 indicated a high variability, which can be attributed to age, sex, breed, and other factors related to the environment, as already highlighted by previous research [45,46]. Nonetheless, the influence exerted by the genetics of the host, reported in humans [47] and livestock [48], should also considered among the factors affecting the gut microbiota.

Several studies have evaluated the variation of salivary cortisol in relation to environment and breed [14] and physical activity [49], but less information is available for the salivary serotonin. Actually, it is known that serotonin in saliva is related to peripheral levels, reflecting circulating plasma concentration and not central serotonin turnover, at least in adult phenylketonuria patients [50]. The results of Table 2 indicate the significant increase of SES in D1 and D3 groups at T28 in comparison to T0. Accordingly, the higher SES concentrations reported above suggest that the serotonin detected in salivary samples derives from host–microbiota interaction.

The activation of the serotonergic nervous system has been reported to decrease the concentration of salivary cortisol in pigs under stressful conditions [51] and, on this basis, a negative correlation between HCS and SES would have been expected. Indeed, SES was significantly higher for D3 and D1 groups at T28 in comparison to the D1 group, even though HCS concentrations were significantly higher at T28 for D0 and D1 groups, suggesting that serotonin in saliva does not probably reflect central serotonergic activity. Bacteria can use tryptophan to produce serotonin [52], and *Escherichia coli*, other than *Lactobacillus* spp., have been reported to be very active. The relative abundance of *Escherichia coli* at T28 was higher in D1 group, (Figure 3), suggesting that the increase of salivary serotonin was related to the presence of this genus, which was almost absent in the D3 group. The increase of salivary cortisol found in D0 and D1 groups could be due to the activation of the HPA from the presence of this gut commensal aero-anaerobic bacillus [52]. However, this consideration deserves further evaluations. According to Mondo et al. (2020) [53], aggressive dogs show a shift of fecal microbiota, with a reduction of Paraprevotellaceae and Mogibacteriaceae. Aggressive dogs also showed an increase of Catenibacterium and Megamonas, but not significant differences in fecal cortisol and testosterone was reported. These results agree with the higher abundances of Paraprevotellaceae and Mogibacteriaceae found in the D3 group, which displayed also the higher SES concentration. Furthermore, the higher abundance of *Fusobacterium* in the D3 group would agree with the results reported in the study of Kirchoff et al. (2019) [54], which reports lower abundance of Fusobacteriaceae in aggressive dogs. Although the results of these two published studies are contradictory for the Paraprevotellaceae, which increased in the latter research, the data would validate a microbiota-gut-brain axis also in dogs.

Metagenomic analysis is a very useful tool to study the dynamics of the microbial populations, but often they do not allow one to quantify microorganisms at genus or species level. For this reason, qPCR analyses were performed in order to focus on some specific genera and species, such as *Faecalibacterium* spp., *Fusobacteria*, *Blautia* spp., *Turicibacter* spp., *Escherichia coli*, *Clostridium hiranonis*, and *Streptococcus* spp., which are some of the most common species and genera normally found in the gut of dogs. Diseases, metabolic disorders, changes in diet, and other factors can interfere with the abundance of these microorganisms in feces [18]. The data obtained from the qPCR analysis indicated that the supplementation of proanthocyanidins in the diet did not determine substantial changes in the composition of the microbial populations analyzed. However, after the administration of the tablets, in all the groups, a high increase in the population of *Streptococcus* spp. was observed. As previously reported, the tablets included barley malt extract, thus, it is possible to speculate that this observed change in the microbiota composition is caused by the inclusion of barley malt extract in the tablets [55].

Several studies investigated the effects of the integration in the diet of polyphenols, finding that some of them are able to promote the adhesion of beneficial bacteria (such as probiotic strains), by inhibiting the colonization of pathogenic microorganisms, such as *Salmonella* spp. and *Escherichia coli*, *S. aureus*, *L. monocytogenes* and *C. albicans* [56]. This could explain the slight reduction in the number of *Escherichia coli* in group D3, after 28 days of treatment, confirmed also by the results obtained from metagenomic analysis. However, there are some parameters that would have been taken into consideration, such as the presence of polyphenols and the products of their microbial metabolism in feces, urine, and blood to better explain their role in microbial modulation.

## 5. Conclusions

The inclusion of proanthocyanidins in the diet of dogs influenced fecal microbiota, and the modifications of relative abundances of the taxa differed between the low and high doses of proanthocyanidins. It is likely that higher doses of proanthocyanidins would be required to induce detectable modifications of fecal microbial community. However, the results highlighted a great variability of relative abundances for all the taxonomic levels among the dogs, suggesting a uniqueness and stability over time of fecal microbiota, which probably responds differently to dietary intervention. Interestingly, salivary biomarkers varied after the inclusion in the diet of proanthocyanidins. Whether the observed variations of salivary serotonin and cortisol are related to the modifications of gut microbiota or to other factors deserves further study.

## Figures and Tables

**Table 1:** Mean concentrations and molar proportions of lactate and volatile fatty acids in the feces of the dogs fed diet without supplementation of grape proanthocyanidins (D0) or supplemented with 1 mg/kg live weight (D1) or 3 mg/kg live weight (D3) of grape proanthocyanidins at the beginning of the study (T0) and after 14 (T14) and 28 (T28) days of administration.

Item	D0			D1			D3			Effects			
	T0	T14	T28	T0	T14	T28	T0	T14	T28	SEM ^1^	Diet	Time	D × T
Lactate (μmol/g)	4.7 ^ab^	3.8 ^ab^	1.4 ^ab^	0.9 ^b^	7.5 ^ab^	2.1 ^ab^	15.3 ^a^	2.5 ^ab^	2.9 ^ab^	1.02	NS	NS	NS
Acetate	143.3	128.8	113.7	137.9	123.4	124.4	143.7	139.6	150.5	4.09	NS	NS	NS
Propionate	39.3 ^b^	35.9 ^b^	38.0 ^b^	39.0 ^b^	49.5 ^ab^	45.5 ^ab^	38.9 ^b^	48.0 ^ab^	61.0 ^a^	1.67	*	*	*
Isobutirate	96.8	61.9	90.5	82.7	58.4	71.0	55.6	72.5	82.2	4.12	NS	NS	NS
Butirate	8.1	8.0	9.8	8.7	11.6	10.6	11.3	11.3	11.1	0.46	NS	NS	NS
Isovalerate	4.2	12.4	4.2	4.5	4.2	4.0	5.3	3.7	5.7	1.06	NS	NS	NS
Total	296.3	250.7	257.6	273.7	254.6	257.6	270.1	277.6	313.3	6.63	NS	NS	NS
Lactate (molar %)	1.8 ^ab^	2.5 ^ab^	0.6 ^b^	0.3 ^b^	6.4 ^ab^	0.8 ^ab^	7.9 ^a^	0.9 ^ab^	1.0 ^ab^	0.75	NS	NS	*
Acetate	48.3	50.8	44.8	50.5	48.4	48.7	52.2	50.2	47.9	0.89	NS	NS	NS
Propionate	13.2 ^b^	14.4 ^ab^	15.0 ^ab^	14.2 ^ab^	18.6 ^ab^	17.8 ^ab^	14.4 ^ab^	17.4 ^ab^	19.6 ^a^	0.52	NS	**	NS
Isobutirate	32.4 ^ab^	24.5 ^abc^	34.0 ^ab^	30.3 ^ab^	19.8 ^bc^	26.9 ^abc^	19.1 ^c^	26.2 ^abc^	26.2 ^abc^	1.19	NS	NS	NS
Butirate	2.8	3.2	3.9	3.1	4.3	4.2	4.4	4.0	3.5	0.17	NS	NS	NS
Isovalerate	1.5	4.7	1.7	1.6	2.5	1.6	2.1	1.3	1.8	0.42	NS	NS	NS

^1^ SEM: standard error of the means. ^a,b,c^: means with different superscripts are significantly different for *p* < 0.05. *: *p* <0.05; **: *p* < 0.01; NS: Not Significant.

**Table 2:** Mean concentrations of cortisol (HCS) and serotonin (SES) in saliva and cortisol in hair (HCH) of dogs fed diet without supplementation of grape proanthocyanidins (D0) or supplemented with 1 mg/kg live weight (D1) or 3 mg/kg live weight (D3) of grape proanthocyanidins at the beginning of the study (T0) and after 28 (T28) days of administration.

Item	D0		D1		D3		Effects			
	T0	T28	T0	T28	T0	T28	SEM ^1^	Diet	Time	D × T
HCS (ng/mL)	1.23 ^a^	4.80 ^a^	1.88 ^ab^	6.26 ^b^	1.37 ^a^	2.88 ^b^	0.44	NS	**	*
HCH (ng/g)	6.80	6.89	6.96	6.56	6.91	7.10	0.15	NS	NS	NS
SES (ng/mL)	32.47 ^b^	34.97 ^b^	42.37 ^ab^	77.64 ^a^	44.31 ^ab^	75.41 ^a^	5.77	NS	**	*
HCS:SES	0.21 ^ab^	0.31 ^a^	0.08 ^b^	0.09 ^ab^	0.07 ^b^	0.05 ^b^	0.03	*	*	NS
HCS:HCH	0.18 ^b^	0.68 ^a^	0.27 ^b^	0.96 ^a^	0.20 ^b^	0.44 ^ab^	0.06	NS	**	NS

^1^ SEM: standard error of the means. ^a,b,c^: means with different superscripts are significantly different for *p* < 0.05. *: *p* < 0.05; **: *p* < 0.01; NS: Not Significant

## Supplementary Materials

The following are available online at https://www.mdpi.com/2076-2615/10/3/531/s1, Table S1: Breed, sex and weight of the dogs recruited for the study.
